# Forecasting Austrian national elections: The Grand Coalition model

**DOI:** 10.1016/j.ijforecast.2013.07.011

**Published:** 2014

**Authors:** Julian Aichholzer, Johanna Willmann

**Affiliations:** a University of Vienna, Department of Methods in the Social Sciences, Rathausstraße 19, 1010 Vienna, Austria; b SUNY at Stony Brook, Department of Political Science, Social and Behavioral Sciences Building, 7th Floor, Stony Brook NY 11794-4392, United States

**Keywords:** Austria, Election forecasting, Economic voting, Multi-party system, Social Partnership

## Abstract

Forecasting the outcomes of national elections has become established practice in several democracies. In the present paper, we develop an economic voting model for forecasting the future success of the Austrian ‘grand coalition’, i.e., the joint electoral success of the two mainstream parties SPOE and OEVP, at the 2013 Austrian Parliamentary Elections. Our main argument is that the success of both parties is strongly tied to the accomplishments of the Austrian system of corporatism, that is, the Social Partnership (*Sozialpartnerschaft*), in providing economic prosperity. Using data from Austrian national elections between 1953 and 2008 (n=18), we rely on the following predictors in our forecasting model: (1) unemployment rates, (2) previous incumbency of the two parties, and (3) dealignment over time. We conclude that, in general, the two mainstream parties benefit considerably from low unemployment rates, and are weakened whenever they have previously formed a coalition government. Further, we show that they have gradually been losing a good share of their voter basis over recent decades.

## 1 Introduction

Forecasts of Austrian national elections have traditionally relied upon classical opinion polls, conducted several days or weeks ahead of an election, or on political stock-markets (Filzmaier et al., 2003, Hofinger and Ogris, 2002). In this paper, we forecast the outcome of the 2013 Austrian parliamentary elections by means of a macroeconomic voting model. While this is established practice in other countries like the US (e.g.  Lewis-Beck and Tien, 2008, Norpoth, 2004), France (e.g.  Foucault & Nadeau, 2012), and Great Britain (e.g.  Lebo and Norpoth, 2011, Lewis-Beck et al., 2004, Sanders, 2005), this kind of forecasting is a novelty to the Austrian case. However, there has been one cross-country comparative forecasting model for radical right parties in Europe, by Evans and Ivaldi (2010), which included Austria as a case. Relying on incumbency, unemployment and the number of asylum seekers, they accurately predicted the vote share of the Austrian Freedom Party (FPOE) in 2008. Another study, by Neck and Karbuz (1997), estimated a popularity function for Austrian parties by drawing upon macroeconomic data (unemployment, inflation, income growth). However, no efforts have been undertaken to model a vote function or to forecast the vote shares of the mainstream Austrian parties based on political and economic indicators.

Thus, our contribution is fourfold. First, we will close this research gap and develop a politico-economic voting model for forecasting the joint success of the two mainstream parties, SPOE (Social Democrats) and OEVP (People’s Party), the so-called ‘grand coalition’, at the 2013 national election. In particular, we ask whether they will manage to keep the absolute majority of votes beyond 2013. Second, we will add new input to the challenges of developing electoral vote forecasts for multiparty systems. The majority of the forecasting models which have been developed have been for two-party or majoritarian systems like the US or Great Britain, where it is common for one single party to form the government, and where it can be determined unambiguously as to who should be held accountable for economic success or failure. Austria, on the other hand, is a multiparty system where various different party coalitions tend to form the government, and where economic accountability is difficult to attribute. Bellucci (2010), Hooghe and Dassonneville (2012), Norpoth and Gschwend (2010), Magalhães and Aguiar-Conraria (2009) and Stegmaier and Lewis-Beck (2009) have already set forth models for various multiparty systems, and have developed individual solutions by drawing upon the respective country-specific circumstances. We will enrich this branch of thinking by developing yet another way of coping with multiparty forecasting. Instead of modeling the vote shares of the individual governing parties, we model the vote share of the grand coalition. In doing this, we are drawing upon the Austrian-specific circumstance of corporatism, arguing that the joint success and economic accountability of the grand coalition parties SPOE and OEVP can be traced back to the way in which they intertwine in their Social Partnership arrangements. Third, we will postulate a parsimonious, politico-economic voting model that gets along without the frequently-used party popularity measure, which is drawn from opinion polls. Thus, we do not rely on opinion polls, but use a model that is created from objective political and macroeconomic data. Fourth, we develop a forecasting model with a comparatively long lead time of up to one year.

The paper proceeds as follows. In Section  2 we will discuss the theoretical underpinnings of our economic voting model and address the peculiarities of the Austrian Social Partnership which help us to overcome the problems in relation to multiparty forecasts. In Section  3, we will specify the details and expectations of our model. Section  4 will elaborate on the data sources used in Section  5, where the voting model is fitted to the past elections. Finally, in Section  6, we will forecast the combined vote share of the SPOE and the OEVP for the 2013 elections. We conclude by summarizing our findings.

## 2 Theory: economic voting and social partnership

When asked about the most important problem facing their country today, many survey respondents indicate that the economy and/or concerns about unemployment are the most important issues to them. A considerable branch of the voting literature, i.e., the economic voting literature (Duch and Stevenson, 2008, Fiorina, 1981, Key, 1966), builds on this pronouncement. This finding has also been confirmed for Austria. More than half of the respondents in the 2009 Austrian National Election Study (56%) indicated that either unemployment or the economy mattered most to them (see  AUTNES, 2009). Beyond the topicality of the economic crisis at this time, these finding are also supported by the findings of other past surveys (see  Müller, 2000, p. 42).

Thus, the state of the economy matters to citizens, and they are comfortable when prosperity is enhanced, economic growth is advanced, and unemployment is decreased. The responsibility hypothesis of economic voting theory assumes that voters reward or punish parties for the state of the economy, that is, economic prosperity or recession, at the poll. It is assumed that they can identify who is responsible for the recent economic ups and downs, and accordingly either support this party at the poll or let it down. This last point poses a problem for the forecasting of vote shares in multi-party systems (Anderson, 2010). In two-party or majoritarian systems, where the government generally consists of only one party, the government’s accountability for the state of the economy can easily be assigned to a single party. However, in multiparty systems where party coalitions frequently form the government, it is still questionable as to which party the voters will hold accountable at the ballot box.

We are by no means the first to recognize that multiparty systems pose a challenge for election forecasting. For instance, Hooghe and Dassonneville (2012) and Norpoth and Gschwend (2010) have already forecasted election outcomes in proportional representative systems. Hooghe and Dassonneville (2012) overcame the multi-party problem by forecasting the vote-share for incumbent parties in general, treating all parties that participated in a coalition as an incumbent party on an equal footing, and assuming that they are all held equally responsible by the voters on election day. This approach requires some sort of repeatedly collected party approval rate measure for leveling out differences in party sizes, which we do not have available for Austria beyond the 1990s. In contrast, Norpoth and Gschwend (2010) met the multiparty challenge using only party-specific variables, no contextual data such as economic well-being. That is, they regress the vote share of every governing coalition only on the characteristics of this very coalition, not on context-specific variables. This, however, is incompatible with our economic vote idea, which assumes that voters blame the incumbent government for *economic* failure or success.

Thus, in order to solve the multiparty challenge of economic voting in Austria, one has to figure out who is seen as responsible for the state of the economy. In other words, who are the voters most likely to hold responsible for economic developments? As Lewis-Beck and Paldam (2000, p.119) put it, “In a multi-party system, the economic voter may target a whole coalition, a party within the coalition, or even assign a particular economic policy to a particular party. Once ‘responsibility’ is properly understood, it can be properly modeled […]”. We argue that, in order to properly understand the responsibility for economic developments in Austria, it is important to consider the actual power over the economy. To a large extent, this power is held within the discretion of the Austrian system of corporatism, that is, the Social Partnership (*Sozialpartnerschaft*).^1^ The Social Partnership, more than any government, has a strong influence on a wide range of economic (and social) questions, and is thus responsible for the success or failure of their policies (Lewis, 2002, Marterbauer, 2005). When it comes to the permanent body of the corporatist consent and its influence on the country’s economic prosperity, Austria is doubtlessly a special case, as has frequently been noted (e.g.  Lewis, 2002). Unlike virtually all other corporatist systems, the influence of the representational organizations of labor and employers in Austria goes well beyond conventional consensus-seeking or policy concertation, and extends to what Tálos and Kittel (2002) term ‘policy accordation’. That is to say, the labor and employer associations not only seek consensus in negotiations with the government, but actually draft policy propositions on a wide range of socioeconomic issues, which are then either submitted directly to the Parliament or passed through the Cabinet to the Parliament. Either way, the propositions remain largely unchanged, and are adopted as suggested by the associations (Tálos, 2005).

Now, one could argue that the Social Partnership has forfeited its influence in the past two decades compared to the ‘golden age’ of the 1960s or 1970s, meaning that its responsibility for the economic well-being of the country would be questionable. Indeed, the increasing internationalization, and in particular the Europeanization, of the economy, leaving national actors with lesser policy discretion, has led authors to speculate about the end of corporatism (Crepaz, 1994, Tálos, 2005). However, we believe that the responsibility assumption still holds, for two reasons. First, the political developments of the 1990s have not necessarily led to an erosion of corporatist power, but rather to a transformation. As Heinisch (2000) points out, the Austrian Social Partnership has fared pretty well in adapting to the altered challenges of internationalization, and rather strengthened its position in the 1990s. He concludes that the corporatist partners have actually become a driving force of Austria’s EU accession, and expanded their reach by diversifying into non-traditional policy fields. Second, and more importantly, formal changes in power do not prevent citizens from believing that the Social Partnership still matters. There is some evidence that popular opinion still sees the Social Partnership as being advantageous for Austria, and citizens tend to expect the institution to exert a fair amount of influence (Heinisch, 2000, Kittel, 2000, Tálos, 2005, pp. 210f; Tálos & Kittel, 2002). This implies that people are aware of the Social Partnership’s functioning and have certain expectations as to its outcome. Thus, they will ascribe responsibility to the Social Partnership both when it delivers economic well-being and when it fails to do so.

Thus, we properly understand the responsibility for economic prosperity as being tied to the Social Partnership. However, this does not immediately help us in effectively *modeling* economic responsibility. After all, the Social Partnership is an extra-parliamentary institution, or what Duch and Stevenson (2008, p. 178) call a ‘nonelectorally dependent decision maker’, which cannot be held directly accountable at the ballot box. However, the institutionalized co-operation is historically very closely linked to the two mainstream Austrian parties, with the OEVP representing the employer organizations and the SPOE representing the employee organizations. Leading representatives of the corporatist associations regularly become Members of Parliament for one of the two parties, and sometimes they are even assigned governmental positions; either way, they are strong opinion leaders within their parties. Thus, the voters can blame or reward the parties for the failures or achievements of the Social Partnership. We assume that voters blame the SPOE and OEVP jointly at the ballot box when economic matters are working unsatisfactorily, and thus, we model the combined vote share of these two parties as a function of the economic prosperity within the country.^2^ As can be seen in Table A.1, the combined vote share of the SPOE and OEVP was very high in the 1960s and 1970s, when the country was experiencing considerable economic growth and economic stability, for which the corporatist system is conventionally held responsible (Gerlich, Grande, & Müller, 1988). With the decelerating economic growth over the subsequent decades, some voters lost their faith in the competence of the SPOE and OEVP and their party-affiliated corporatist organizations in directing the country’s economy to sufficient economic prosperity, and the combined vote share decreased accordingly.

## 3 Specifying the model

After having made a case for the basic logic of our model, i.e., the SPOE and OEVP are jointly held responsible for economic failure and success, we will now specify it in more detail. Specifically, our independent variables are unemployment, incumbency, and a measure for progressing partisan dealignment.

We build our model upon *unemployment* as the main economic measure, for two reasons. On the one hand, we contend that the unemployment rate is a good indicator of the general economic prosperity within a country. This measure frequently fares well in forecast models. While Lewis-Beck and Tien (2005), for example, use jobs in their (Jobs) model for the US, Arzheimer and Evans (2010) and Foucault and Nadeau (2012) draw upon unemployment as an economic indicator for forecasting French national elections, as do Magalhães, Aguiar-Conraria, and Lewis-Beck (2012) for Spanish elections. Second, we make a case on the voters’ individual evaluations. As our argument is that people blame the SPOE and the OEVP for the success of the institutionalized employer/employee co-operation, it seems reasonable to choose an indicator which the Social Partnership influences. Furthermore, as we have seen above, people consider unemployment to be a pressing issue—not only those who are directly concerned by it, but also those who actually have jobs. When jobs are scarce, wages are not raised and it is difficult to change employment positions so as to build one’s career. Thus, people generate a general bad mood when talking among themselves about bad economic prospects, and we assume that voters follow this general mood and weaken the two mainstream parties as unemployment increases.

To sum up, we will rely on the general unemployment rate as an indicator of economic prosperity, and we expect the relationship between the unemployment rate and the support for the mainstream parties to be negative: the higher the unemployment rate, the fewer votes will be cast for the grand coalition and the more protest votes will be gained by other (opposition) parties. We argue that the causal link is primarily through the Social Partnership; that is, the parties which represent employers and employees are rewarded or punished by the voter for the state of the economy. However, it seems reasonable to assume that this effect will matter more (or only) if both parties were incumbent in the previous term and formed a so-called ‘grand coalition’. If one of the two parties was an opposition party, it could recover and regain votes from the other party again. Technically speaking, we will therefore interact the unemployment rate with a variable indicating grand coalition incumbency.^3^

Further, we argue that *incumbency* of the two parties itself can have a negative effect on vote shares. We argue that the popularity of the coalition parties dwindles when they are in office, what Norpoth (1991) terms ‘incumbency fatigue’. In particular, their practice of the *Proporz* (proportional) system is assumed to tire out the voters. Jobs in nationalized industries and administration, including the public service broadcaster (ORF), are usually shared among the SPOE and the OEVP (e.g.  Ennser-Jedenastik, 2013). This practice has often been criticized as intransparent, unjust, and inflexible, and may therefore harm their success in the subsequent election. Note, however, that we do not make specific hypotheses about the leading party in a grand coalition government. That is, we implicitly assume equality in the attribution of responsibility. Further, when looking at our interaction hypothesis, we have good reason to believe that there should be no impact of incumbency if unemployment rates approach zero.

Finally, we have to make allowances for the steady *partisan dealignment* (see  Dalton & Wattenberg, 2000) in our model, which has been affecting the baseline vote share of the two mainstream parties since the 1950s. In other words, it is very unlikely that, *ceteris paribus*, the two parties will gain the same vote shares today as they did in the 1960s or 1970s. While Austria used to be characterized by high rates of party membership and a strong party identification with the two mainstream parties, this phenomenon has gradually decreased over time. Since we lack reliable and updated data on issues like party membership figures, voter survey data on partisan affect, or socio-structural changes in classical cleavage groups in the constituency, we insert a time index variable to capture the dealignment effect (see for example  Fair, 1988). Moreover, a time index is often used for detrending time series data.

**Fig. 1:**
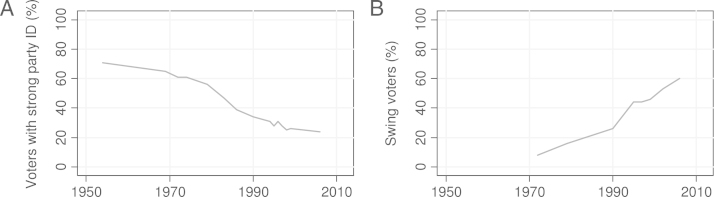
Indicators of partisan dealignment in Austria.

Though our time index is a rather rough indicator, it serves as a proxy for otherwise unavailable data and can be seen as the ‘common factor’ underlying several indicators of dealignment. Indeed, the available figures (see Fig. 1) show that party identification in Austria has been declining since the mid-1950s, while swing voting is on the rise. Thus, several social changes in the constituency suggest that there has been a severe and continuous decline in the voter basis of both the SPOE and the OEVP that is still ongoing. It is not yet clear when this decline will reach a preliminary bottom. Hence, once the pattern in decline changes (at some stage in the future), forecasting with the present model will become inaccurate.

Müller, Plasser, and Ulram (1999, p. 206); Plasser, Ulram, and Seeber (2007, p. 169).

## 4 Data

We have compiled data for the past eighteen parliamentary elections, going back to the election of 1953 (n=18) and covering the elections of 1953, 1956, 1959, 1962, 1966, 1970, 1971, 1975, 1979, 1983, 1986, 1990, 1994, 1995, 1999, 2002, 2006 and 2008. As has already been mentioned, we include macroeconomic indicators as well as political and institutional factors, as is frequently done in economic voting forecasting models (Campbell, 2012, Lewis-Beck, 2005). We use data on the electoral success of the parties, as well as incumbency, which is taken from the official bulletin of the Austrian Ministry of the Interior (BMI).^4^ Unemployment rates are provided by the Austrian National Bank (OeNB).^5^ Since we use the unemployment rate as our main independent variable for economic voting, we are limited by the unavailability of this indicator before the 1950s. However, this is not really a problem, in the sense that the two post-WWII elections (1945 and 1949) represent rather atypical cases. The unemployment rate, i.e., the annual average, is measured with a lag time of one year before each election. We thus assume that the economic conditions of the past year will have a lagged impact on future vote choices. We do not consider the development over a longer period, since voters are usually assumed to have relatively short time horizons with regard to economic evaluations (see  Lewis-Beck & Paldam, 2000).

In our model, we finally include the combined vote share of the two parties as our dependent variable (M=80.95, S.D.=12.90, Min=55.24, Max=93.37), together with the unemployment rate in the year before the election (y−1) (M=4.77, S.D.=2.00, Min=1.50, Max=7.50), an interaction of unemployment and a joint incumbency of SPOE and OEVP in the previous election (incumbent in 56% of the elections), as well as a time index variable (see for example  Fair, 1988). The time index variable acts as a control for the assumed dealignment process and the increased volatility of the electorate at each election. We also tried to model the loss of the mainstream parties’ voter basis using other proxies for long-term changes in partisanship, such as a moving average of the lagged vote shares of previous elections, as was suggested by Norpoth and Gschwend (2010). However, the lagged vote shares follow a curvilinear pattern that goes hand in hand with low unemployment rates in the 1970s, and hence, cannot be considered an independent trend. Rather, an inspection of the model residuals suggests that there is an almost linear, time-dependent trend (serious autocorrelation) in vote shares that is explained by neither unemployment nor incumbency. Nevertheless, it is important to note that our time index variable serves only as an approximation for a societal trend which we can observe for the previous elections, and which we assume will also be true for the next election. Hence, we do not encourage the making of predictions based on this index variable for the infinite future, as this will result in a natural extinction of the two parties. Rather, this trend must be updated with each future election.

**Fig. 2:**
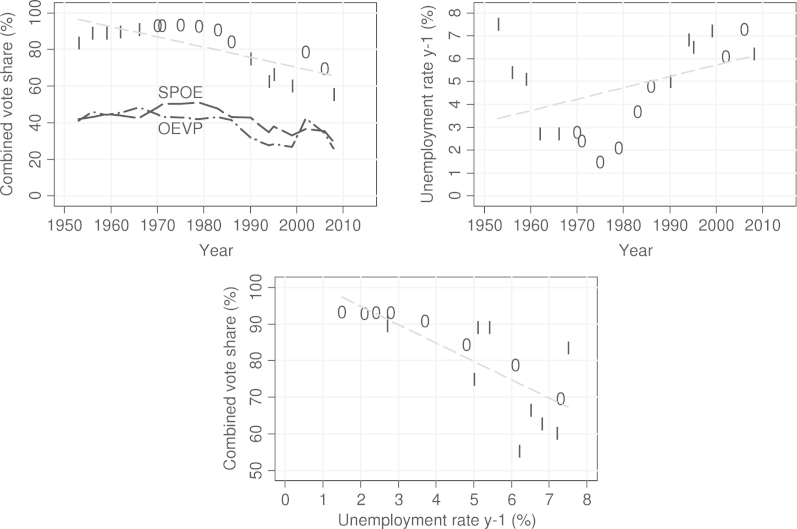
Scatterplots for independent variables in the model (1 = incumbent, 0 = not incumbent).

In order to achieve familiarity with the data, we provide bivariate scatterplots (Fig. 2) for the main variables in our model for all of the election years under consideration. Note that the markers represent the dummy variable on the previous incumbency (1 =  incumbent, 0 =  not incumbent) of both parties.

In the graph, we see several important relationships using bivariate associations: the combined vote share has been decreasing since the mid-1970s, though in a curvilinear shape, while the unemployment rate was relatively high in the 1950s and 1960s, was very low in the mid-1970s, and has been increasing again since the 1980s. Also, most importantly, vote shares are lower at higher unemployment rates. Finally, there is a certain pattern of lower vote shares when both parties were previously the incumbents.

## 5 Models and results

It is common practice to require a forecasting model to satisfy four main criteria: *lead, parsimony, accuracy,* and *reproducibility*  (Lewis-Beck, 2005). In what follows, we will address each of these points in turn. Since our aim is to look into the future, our forecasting model must produce good estimates at a considerable *lead* time. Thus, we use the average unemployment rate of the year preceding an election.^6^ Compared to other forecast models, this is an unusually long lead time, with predictions being possible up to one year in advance (see  Lewis-Beck, 2005, p.157). In terms of *parsimony*, we try to use as few independent variables as possible while fitting the forecast accurately. The respective data can be found in the Appendix, to enable the model to be *reproduced*. Finally, the *accuracy* of a forecast will be discussed in detail in the following section.

In what follows, we estimate the combined vote share of SPOE and OEVP using our predictor variables for the elections from 1953 till 2008 (n=18). We estimate five separate models, each time using ordinary least squares (OLS) regressions. The final model, which we use for making the forecast, consists of the unemployment rate of the year before the election (U), the joint incumbency of SPOE + OEVP (I), and an election index variable (E), with each election being a discrete event in time (where 1953 =  0, the first election in our data set). To cover the interaction hypothesis mentioned earlier, we include an interaction term (U⋅I) which states that the effect of the unemployment rate on the parties’ vote share is moderated by their previous incumbency. Hence, our regression equation reads as follows:

(1) $$\overset{ˆ}{V}=\beta_{0}+\beta_{1}\cdot U+\beta_{2}\cdot I+\beta_{3}\cdot E+\beta_{4}\cdot U\cdot I\text{.}$$

We compare the models and check whether the model fit improves significantly when introducing independent variables using classical fit measures. Table 1 below provides several indicators for the accuracy of the estimated model. The $R^{2}$ estimate gives the percentage of the variance which is accounted for by the predictor variables, whereas the adjusted $R^{2}$ is corrected for the sample size and the number of predictors, and is thus a more conservative model fit measure. Since the elections are not a sample in the traditional sense, the improvement in prediction $\left(R^{2}\right)$ is regarded as a more suitable indicator of a variable’s explanatory power than the standard errors of regression coefficients. The Standard Error of Estimates (SEE) is seen as a good estimate of the average error in a prediction, and is usually used to calculate a confidence interval of the forecast (see  Lewis-Beck, 2005, p. 153). We also report the mean absolute error (MAE) of the forecast residuals. Lower AIC and BIC values indicate a relatively better fit of the model to the data. Finally, the Durbin–Watson (D–W) statistic informs us about issues of autocorrelation in the time series data.

**Table 1:** Forecast model estimates of the combined vote share and fit measures.

OLS Estimates	Model 1	Model 2	Model 3	Model 4	Model 5	Final model
	b (S.E.)	b (S.E.)	b (S.E.)	b (S.E.)	b (S.E.)	b (S.E.)
Unemployment rate (y−1)	−5.02⁎⁎⁎⁎			−1.88⁎⁎⁎	−0.96⁎	−0.82
	(1.01)			(0.49)	(0.52)	(0.47)
Incumbency SPOE-OEVP		−10.96⁎		−10.91⁎⁎⁎⁎	−2.23	
		(5.68)		(1.76)	(3.44)	
Election no. (index 1953 = 0)			−1.84⁎⁎⁎⁎	−1.71⁎⁎⁎⁎	−1.74⁎⁎⁎⁎	−1.73⁎⁎⁎⁎
			(0.39)	(0.17)	(0.14)	(0.14)
Unemployment ⋅ Incumbency					−1.86⁎⁎	−2.26⁎⁎⁎⁎
					(0.67)	(0.28)
Constant term	104.87⁎⁎⁎⁎	87.04⁎⁎⁎⁎	96.55⁎⁎⁎⁎	110.53⁎⁎⁎⁎	107.28⁎⁎⁎⁎	106.52⁎⁎⁎⁎
	(5.19)	(4.23)	(3.91)	(1.97)	(1.99)	(1.59)

$R^{2}$	0.61	0.19	0.58	0.95	0.97	0.97
Adj. $R^{2}$	0.58	0.14	0.55	0.94	0.96	0.96
SEE	8.32	11.97	8.64	3.03	2.49	2.44
MAE	5.99	9.95	7.23	2.23	1.84	1.80
AIC	129.25	142.34	130.61	94.48	88.05	86.63
BIC	131.03	144.12	132.39	98.04	92.51	90.19
D–W statistic	0.83	0.23	0.82	2.27	2.39	2.19
n	18	18	18	18	18	18

Note: Two-tailed significance.

⁎ p<0.10.

⁎⁎ p<0.05.

⁎⁎⁎ p<0.01.

⁎⁎⁎⁎ p<0.001.

First, we ran a regression to test our model with regard to party-specific effects (see Table A.2 in the Appendix). Overall, the results tell us that the model works well for predicting the combined vote share of both parties, and also if we examine SPOE and OEVP incumbency and its interaction with unemployment separately. We demonstrate that the total vote share is particularly diminished whenever both parties are incumbent. However, we expect an interaction effect to be at work. Note that, in interaction models, the main effects of one variable (x1) can be read as the average effect if the other main variable (x2) was zero (for a discussion, see  Brambor, Clark, & Golder, 2006). To prevent inefficiency (variance inflation) due to collinearity, we omit the main effect of incumbency in the interaction model (β${}_{2}=0$), as there is strong theoretical reason and empirical support to believe that there is no effect when the unemployment approaches zero. We show that the unemployment effect reaches its maximum if both parties were incumbent (sum of interaction effects). At the same time, if only one of the two parties was in power, unemployment has hardly any effect on the combined vote share, as all of the effects basically balance out (main effect plus one interaction effect). Since the interaction effects of incumbency are statistically indistinguishable, we proceed with the intended, and more parsimonious, model using the shared incumbency.^7^

Next, we start out by estimating separate regression analyses for our full model, i.e., the combined vote share, in order to examine bivariate results as well (see Table 1). We see that unemployment (Model 1), incumbency (Model 2), and the election index, i.e., going from one election to the next (Model 3), all have a negative effect on the combined vote share of the SPOE and OEVP, though other factors are not controlled for. In particular, more than half of the variance is accounted for by either unemployment or a hypothesized linear trend in dealignment. The effects of unemployment, incumbency, and the election index also remain important after controlling for each other (Model 4). That is, higher unemployment rates (around −2%), having formed a grand coalition in the previous election (around −11%), and a loss in the baseline vote share in each consecutive election (around −2%) will all harm the two parties. As has been mentioned in previous sections, we would expect the effect of unemployment to weigh more heavily if SPOE and OEVP were the incumbent parties (Model 5). A variable representing this interaction adds further explanatory power, and confirms that unemployment rates will especially harm the parties governing a coalition. Again, we see that incumbency has no effect in this model if the unemployment rate approaches zero. Still, we find some indication that higher unemployment rates would have only a minor negative effect on the combined vote share of the SPOE and the OEVP if they were not both incumbent. We omit the main effect of incumbency (β${}_{2}=0$) in our final model to be used for an accurate forecast (see the final model), also for theoretical reasons. In doing so, we get more efficient estimates (we reduce the collinearity due to the interaction terms) and a better model fit.^8^ The final model performs very well according to all of the fit and accuracy measures, showing that a combination of high unemployment rates and being incumbent (interaction term), together with a continuous decline in partisan support, will reduce the combined vote share of the two mainstream parties SPOE and OEVP.

In order to verify that we have estimated the actual vote share reasonably well, we compare the actual vote share to the model estimates, also using out-of-sample errors, which occur if observations (i.e. elections) are consecutively omitted from the data set (see Table 2). Both the values for Cook’s D (D=0.69), i.e., the influence on the overall regression results, and the Dfbeta statistic, i.e., the change in parameters when one case is excluded, suggest that the 2006 election is somewhat troublesome. The residuals (S.D.=2.21, Min=−3.57, Max=3.31) indicate that the largest estimate is 3.57% off the actual result. In particular, we see that the model performs worst for the 1962 election. In general, the model also performs somewhat worse in the aftermath of the coalition of OEVP and FPOE (Freedom Party of Austria) (elections 2002 and 2006), with the latter suffering from the secession of the newly founded party BZOE (Alliance for the Future of Austria) at this time.

**Table 2:** Comparison of actual vote shares and model estimates (final model).

Year	V	$\overset{ˆ}{V}$	e	*oof*	*ooe*
2008	55.24	57.94	−2.70	59.30	−4.06
2006	69.67	72.78	−3.11	75.57	−5.91
2002	78.81	75.50	3.31	74.17	−2.54
1999	60.06	60.06	0.00	60.06	−0.00
1995	66.35	63.95	2.40	63.42	2.93
1994	62.59	64.76	−2.17	65.21	−2.62
1990	74.84	72.04	2.80	71.63	3.21
1986	84.42	85.24	−0.82	85.39	−0.97
1983	90.87	87.87	3.00	87.49	3.38
1979	92.93	90.92	2.01	90.50	2.43
1975	93.37	93.15	0.22	93.09	0.28
1971	93.15	94.15	−1.00	94.31	−1.16
1970	93.11	95.55	−2.44	95.95	−2.84
1966	90.91	91.27	−0.36	91.33	−0.42
1962	89.43	93.00	−3.57	93.66	−4.23
1959	88.98	87.34	1.64	86.99	1.99
1956	89.00	88.15	0.85	87.89	1.11
1953	83.37	83.42	−0.05	83.46	−0.09

Note: Actual vote share (V), estimated vote share from the model ($\overset{ˆ}{V}$), estimation residuals (e), out-of-sample forecast (oof), and out-of-sample error (ooe).

**Table A.1:** Vote shares of the two main parties and model variables.

Election	SPOE	OEVP	V	U	I-SPOE	I-OEVP	I (both)	E
2008	29.26	25.98	55.24	6.2	1	1	1	17
2006	35.34	34.33	69.67	7.3	0	1	0	16
2002	36.51	42.30	78.81	6.1	0	1	0	15
1999	33.15	26.91	60.06	7.2	1	1	1	14
1995	38.06	28.29	66.35	6.5	1	1	1	13
1994	34.92	27.67	62.59	6.8	1	1	1	12
1990	42.78	32.06	74.84	5.0	1	1	1	11
1986	43.12	41.30	84.42	4.8	1	0	0	10
1983	47.65	43.22	90.87	3.7	1	0	0	9
1979	51.03	41.90	92.93	2.1	1	0	0	8
1975	50.42	42.95	93.37	1.5	1	0	0	7
1971	50.04	43.11	93.15	2.4	1	0	0	6
1970	48.42	44.69	93.11	2.8	0	1	0	5
1966	42.56	48.35	90.91	2.7	1	1	1	4
1962	44.00	45.43	89.43	2.7	1	1	1	3
1959	44.79	44.19	88.98	5.1	1	1	1	2
1956	43.04	45.96	89.00	5.4	1	1	1	1
1953	42.11	41.26	83.37	7.5	1	1	1	0

Notes: V=combined vote share, U=unemployment rate  (y−1), I=previous incumbency, and E=index of election.

**Table A.2:** Forecast model estimates for the combined vote share (incumbency effect estimated separately).

OLS estimates	Model 1	Model 2	Model 3	Model 4
	b (S.E.)	b (S.E.)	b (S.E.)	b (S.E.)
Incumbency SPOE	−4.45	−11.60⁎⁎⁎⁎	0.68	
	(7.77)	(2.19)	(5.46)	
Incumbency OEVP	−14.87⁎⁎	−10.17⁎⁎⁎⁎	−2.52	
	(6.47)	(2.24)	(4.36)	
Unemployment rate (y−1)		−2.00⁎⁎⁎	1.41	1.73⁎
		(0.55)	(1.41)	(0.81)
Election no. (index 1953 = 0)		−1.71⁎⁎⁎⁎	−1.76⁎⁎⁎⁎	−1.74⁎⁎⁎⁎
		(0.17)	(0.15)	(0.14)
Unemployment ⋅ Incumbency SPOE			−2.29⁎	−2.16⁎⁎⁎⁎
			(0.97)	(0.31)
Unemployment ⋅ Incumbency OEVP			−1.92	−2.53⁎⁎⁎⁎
			(1.12)	(0.46)
Constant term	95.40⁎⁎⁎⁎	122.05⁎⁎⁎⁎	106.88⁎⁎⁎⁎	105.87⁎⁎⁎⁎
	(9.39)	(3.10)	(6.30)	(1.84)

$R^{2}$	0.26	0.96	0.97	0.97
Adj. $R^{2}$	0.16	0.94	0.96	0.96
SEE	11.80	3.11	2.64	2.48
MAE	8.94	2.25	1.79	1.80
AIC	142.67	96.04	91.13	87.87
BIC	145.34	100.50	97.37	92.32
D–W statistic	0.39	2.24	2.58	2.50
n	18	18	18	18

Note: Two-tailed significance.

⁎ p<0.10.

⁎⁎ p<0.05.

⁎⁎⁎ p<0.01.

⁎⁎⁎⁎ p<0.001.

## 6 Making the forecast

We move on to making a forecast for the 2013 election (the 18th election, according to the time index variable), using current labor market data. The annual average unemployment rate for the year 2012 was 7.0% (source: OeNB). Drawing upon this scenario and including the current incumbency of the two parties, we substitute the values into our regression equation (Eq. (1)) as follows:

(2) $$\overset{ˆ}{V}=\text{106.52 }-0.82\cdot 7+0-\text{1}.73\cdot 18-2.26\cdot 7\cdot 1=\text{5}3\text{.74 .}$$

**Fig. 3:**
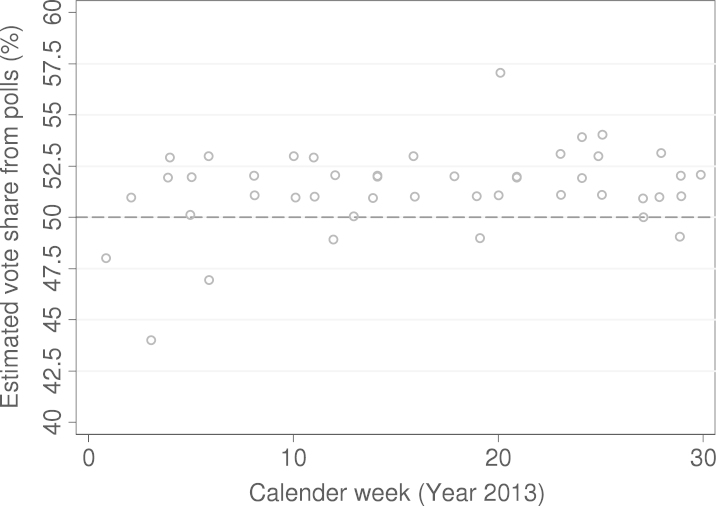
Forecasts of vote shares according to polls.

According to the final model, the two parties will gain about 54% of the popular vote share in the coming election. This is somewhat higher than current polls (since the beginning of 2013) suggest (Fig. 3), as they predict that the combined vote share will be around the 50% mark.

The data are from the polling institutes Gallup, Karmasin, Market, Hajek, IMAS, Oekonsult, Spectra, and meinungsraum.at (n=45, M=51.38, S.D.=2.03, Min=44, Max=57) (last entry: 26 July 2013).

We also provide estimates of the uncertainty of our prediction. First, we provide 95% confidence intervals (CI), which are computed from the SEE times the t-value at given degrees of freedom:

(3) $$CI=\overset{ˆ}{V}\mp SEE\cdot t_{df=14}=\overset{ˆ}{V}\mp 2.44\cdot 2.145\text{.}$$

According to this calculation, the interval states with 95% confidence that the combined vote share of the two parties will be between 49% and 59% in the next election.

**Fig. 4:**
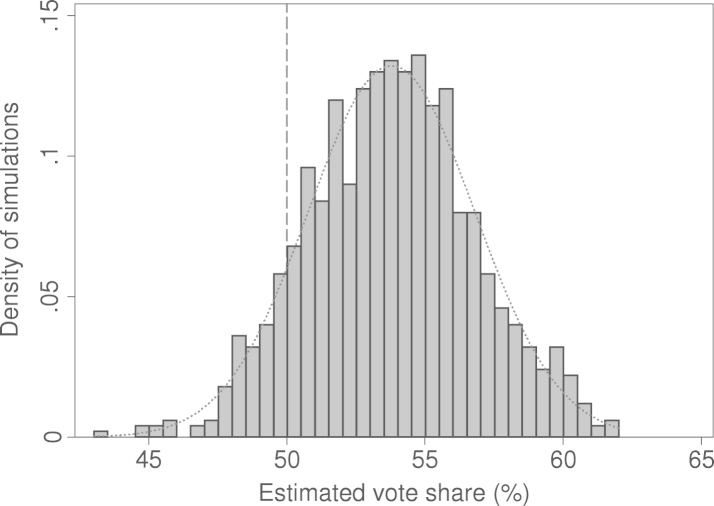
Histogram of simulated expected vote shares (n=1000 simulations).

Second, we use simulations to describe the results of our statistical model. As was pointed out by King, Tomz, and Wittenberg (2000), the estimates of the regression parameters are not perfectly certain, but show fluctuations and stochastic uncertainty. We can, however, calculate forecasts using the point estimates and the variance-covariance matrix of the estimates in our model. King et al. (2000) show how this can be done.^9^ The main difference in the simulation model is that it uses exact values for each independent variable (see Eq. (2)) rather than a general uncertainty measure, such as the SEE. We use 1000 simulations to compute the expected values that incorporate these uncertainty components. Fig. 4 shows the density (distribution) of these expected values.

The predicted value for our forecast is equal to the average of the expected predictions, i.e., roughly 53.7%. The uncertainty range (95% CI) shows a lower bound of 50.5% and an upper bound of 56.8%, which is somewhat smaller than that estimated by means of the SEE method. We can also use the simulation results to get a better picture of the uncertainty of this forecast. 90% of the simulations generated a value >50, which gives us the approximate probability that the vote share will actually be larger than 50% (see  King et al., 2000, p. 349), using the values of our independent variables given in Eq. (2).

## 7 Conclusion

In this paper, we develop an economic voting forecast model for the Austrian case. We basically draw upon the unemployment rate and incumbency, as we lack a measure of party popularity with which to forecast the coming elections. For this purpose, therefore, we used the unemployment rate with a long lead time of up to one year, which allows us to predict the vote shares quite accurately. Still, this is a quite extensive lead time, and future research might investigate the impact of time in economic developments on voting in more depth.

Furthermore, we overcome the challenges which are usually connected with multiparty forecasts by predicting the *joint* vote share of the grand coalition parties SPOE and OEVP. We argue that those parties will be held accountable at the polls for economic ups and downs, as they are the political representation of the Social Partnership. That is, we maintain that when the economy prospers, voters will be satisfied with the performance of the corporatist organizations, and reward the two mainstream parties. This effect is apparently stronger if the parties were incumbent in the previous term, but is also traceable to a very small degree if they were not.

According to our forecast, the next election will be very close, with the combined vote share of the mainstream parties SPOE and OEVP being only 3.7 points above the 50% mark. We are also highly confident that they will not be able to reach the two-thirds majority which would enable them to pass laws of constitutional status. At the same time, we have to emphasize that a 50% majority does not necessarily mean that the two parties ultimately will, or indeed will be able to, form a governmental coalition. This, of course, also depends on their political will and the translation of vote shares into seats in parliament, which in turn will be affected by the vote shares of the smaller parties that do or do not reach the threshold necessary for representation in Parliament.

Finally, we discuss some limitations. As is obvious, the model uses a very rough measure of the ‘natural’ decline in party support of the two mainstream parties since the 1950s. A more exact measure of the actual party support is desirable in order to be able to cope with future changes in the pattern of partisan dealignment. Furthermore, our model depends on the assumptions that the Social Partnership is perceived by the public as a decisive actor in socioeconomic policy-making, and that the SPOE and OEVP are held jointly responsible at the poll for the success or failure of ‘policy accordation’. This conjecture has not gone unchallenged (see  Karlhofer & Tálos, 2005), and our model may lose in predictive power as the Social Partnership loses in political power. Notwithstanding this, we have shown that lagged unemployment rates perform very well in explaining the results of past elections since 1953. Thus, both mainstream parties make ground on low unemployment rates, and thus depend on each other.

## Appendix

See Table A.1, Table A.2.

## Footnotes

1 This institutionalized cooperation consists of representatives from the Trade Union Federation (ÖGB), the Federal Economic Chamber (WKÖ), the Federal Chamber of Labour (BAK), and the Chamber of Agriculture (LK). See http://www.sozialpartner.at/sozialpartner/Sozialpartnerschaft_mission_en.pdf.

2 Note that, looking at the past 18 elections (1953–2008), the vote shares of the SPOE and the OEVP have been relatively closely correlated (r=0.74).

3 See the data on incumbency in Table A.1 in the Appendix. Besides one-party governments (elected 1966–1979), there were four other coalitions, namely SPOE/FPOE (elected 1983, 1986), and OEVP/FPOE (elected 1999, 2002). However, it has never occurred that neither of the two parties participated in the government.

4 http://www.bmi.gv.at/cms/BMI_wahlen.

5 http://www.oenb.at/en/stat_melders/datenangebot/datenangebot.jsp.

6 We also checked whether deseasonalized unemployment rates (with a constant seasonal pattern) would be more appropriate, using various different lag times (2–6 months before each election). However, the annual unemployment rate one year before each election produced the most accurate prediction.

7 Note that using the model including party-specific incumbency gives basically the same forecast results.

8 We stick to omitting the constitutive term of incumbency, as the regression coefficient estimates and forecast results hardly change. Again, model fit measures support the usefulness of this final model for producing the forecasts.

9 We use the STATA package ‘clarify’, by King et al. (2000), to calculate our predictions.
